# Cardiopulmonary exercise testing for identification of patients with hyperventilation syndrome

**DOI:** 10.1371/journal.pone.0215997

**Published:** 2019-04-23

**Authors:** Kristian Brat, Nela Stastna, Zdenek Merta, Lyle J. Olson, Bruce D. Johnson, Ivan Cundrle

**Affiliations:** 1 Department of Respiratory Diseases, University Hospital Brno, Brno, Czech Republic; 2 Faculty of Medicine, Masaryk University, Brno, Czech Republic; 3 Department of Cardiovascular Diseases, Mayo Clinic, Rochester, MN, United States of America; 4 Department of Anesthesiology and Intensive Care, St. Anne´s University Hospital, Brno, Czech Republic; 5 International Clinical Research Center, Brno, Czech Republic; Plymouth State University, UNITED STATES

## Abstract

**Introduction:**

Measurement of ventilatory efficiency, defined as minute ventilation per unit carbon dioxide production (V_E_/VCO_2_), by cardiopulmonary exercise testing (CPET) has been proposed as a screen for hyperventilation syndrome (HVS). However, increased V_E_/VCO_2_ may be associated with other disorders which need to be distinguished from HVS. A more specific marker of HVS by CPET would be clinically useful. We hypothesized ventilatory control during exercise is abnormal in patients with HVS.

**Methods:**

Patients who underwent CPET from years 2015 through 2017 were retrospectively identified and formed the study group. HVS was defined as dyspnea with respiratory alkalosis (pH >7.45) at peak exercise with absence of acute or chronic respiratory, heart or psychiatric disease. Healthy patients were selected as controls. For comparison the Student t-test or Mann-Whitney U test were used. Data are summarized as mean ± SD or median (IQR); p<0.05 was considered significant.

**Results:**

Twenty-nine patients with HVS were identified and 29 control subjects were selected. At rest, end-tidal carbon dioxide (P_ET_CO_2_) was 27 mmHg (25–30) for HVS patients vs. 30 mmHg (28–32); in controls (p = 0.05). At peak exercise P_ET_CO_2_ was also significantly lower (27 ± 4 mmHg vs. 35 ± 4 mmHg; p<0.01) and V_E_/VCO_2_ higher ((38 (35–43) vs. 31 (27–34); p<0.01)) in patients with HVS. In contrast to controls, there were minimal changes of P_ET_CO_2_ (0.50 ± 5.26 mmHg vs. 6.2 ± 4.6 mmHg; p<0.01) and V_E_/VCO_2_ ((0.17 (-4.24–6.02) vs. -6.6 (-11.4-(-2.8)); p<0.01)) during exercise in patients with HVS. The absence of V_E_/VCO_2_ and P_ET_CO_2_ change during exercise was specific for HVS (83% and 93%, respectively).

**Conclusion:**

Absence of V_E_/VCO_2_ and P_ET_CO_2_ change during exercise may identify patients with HVS.

## Introduction

Hyperventilation syndrome (HVS) is characterized as episodic dyspnea with inappropriately high alveolar ventilation exceeding metabolic requirements [1,2]. HVS is highly prevalent in patients with psychological pathologies [3]. However, it is not clear if psychological pathologies are a cause of HVS [4,5].

There are no clear diagnostic criteria or screening tools for HVS [6] and the diagnosis is typically made by exclusion of other cardiopulmonary diseases characterized by symptoms of dyspnea including heart failure, asthma or chronic obstructive pulmonary disease [7]. Previously, the hyperventilation provocation test was used to identify patients with HVS [1]. However, this test has been considered invalid and is no longer used in clinical practice [8,9]. Questionnaires (especially the Nijmegen questionnaire [10]) have also been used to assess symptoms typical for HVS. However, it has been recommended that the Nijmegen questionnaire no longer be used as the sole criterion for HVS and a multidimensional diagnostic approach is advised [11].

Markers of hyperventilation including increased minute ventilation per unit of carbon dioxide production (V_E_/VCO_2_) and low partial pressure of end-tidal carbon dioxide (P_ET_CO_2_) during exercise have been shown to be associated with HVS [12,13]. The slope of V_E_/VCO_2_ is increased in other conditions which need to be distinguished from HVS [14] which may limit this marker as a screening tool for HVS. However, cardiopulmonary exercise testing (CPET) is useful for evaluation of the differential diagnosis of dyspnea [15] suggesting patients with HVS may benefit from this evaluation. A more specific marker for HVS would be clinically useful.

We hypothesized that ventilatory control is abnormal at rest and during exercise in patients with HVS. Accordingly, the aim of the study was to compare CPET of subjects with HVS and healthy controls in order to identify rest or exercise CPET parameters which may be useful for the diagnosis of HVS.

## Methods

### Subject selection

Medical records of all individuals that underwent CPET at the Department of Respiratory Diseases, University Hospital Brno between January 1^st^, 2015 and December 31^st^, 2017 were retrospectively analyzed. HVS was defined similar as in previous studies [6,7]; episodes of dyspnea with documented respiratory alkalosis (pH >7.45) by arterial blood gas analysis at peak exercise and the absence of known acute or chronic respiratory, heart or psychiatric disease. Controls matched by pulmonary function testing were selected from healthy subjects that completed CPET at the Department of Respiratory Diseases, University Hospital Brno between January 1^st^, 2017 and December 31^st^, 2017. The study was approved by the institutional Ethics Committee of the University Hospital Brno, Czech Republic (study approval code 01–070318).

### Cardiopulmonary exercise testing

All subjects underwent symptom-limited CPET on an electronically braked cycle ergometer (Ergoline, Ergometrics 800, Germany) with a 12-channel electrocardiography unit (Schiller AG, AT-104, Switzerland) using a ramp protocol with linear increase of workload of 25 watts per minute. Expired gases were collected and analyzed by the PowerCube-Ergo system (Ganshorn Medizin Electronic GmbH, Germany). Arterial blood gases were examined at rest and at peak exercise. The measured spiroergometric variables included oxygen consumption (VO_2_), output of carbon dioxide (VCO_2_), partial pressure of end-tidal carbon dioxide (P_ET_CO_2_), tidal volume (V_T_), breathing frequency (f_b_) and minute ventilation (V_E_). The data were recorded continuously. The variables were reported as average values obtained during the final 30 seconds of each workload. The derived parameters included respiratory exchange ratio (RER), defined as the ratio of VCO_2_ and VO_2_, dead space volume to tidal volume ratio (V_D_/V_T_), V_E_/VCO_2_ slope and V_E_/VCO_2_ ratio for rest and peak exercise [16]. V_T_ was indexed to body surface area (BSA) [17] to allow comparison between groups (there was a significant difference in sex between both groups).

### Pulmonary function tests

Spirometry was performed in all subjects before CPET. All measurements were performed in accordance with the American Thoracic Society standards [18] using the ZAN 100 spirometer (nSpire Health, Inc., Longmont, CO, USA). The following variables were considered for further assessment: forced vital capacity (FVC), forced expiratory volume in 1 second (FEV_1_) and the FEV_1_/FVC ratio. The values of FEV_1_ and FVC were expressed as a percentage of predicted value.

### Statistics

The Shapiro-Wilk test was used to evaluate normality. Student t-test or Mann-Whitney U test were used for comparison of subjects with HVS and controls. ANOVA and Tukey's HSD post-hoc test was used to evaluate changes of V_E_/VCO_2_ and P_ET_CO_2_ during exercise. Linear regression was performed to evaluate the relationship between P_ET_CO_2_ or V_D_/V_T_ and V_E_/VCO_2_ ratio at peak exercise. Differences in proportions were tested by two-tailed Fisher exact test. Logistic regression analysis adjusted for potential confounders (subject characteristic parameters which were significantly different between both groups–gender and BMI) was used to evaluate the difference of (peak-rest) P_ET_CO_2_ and difference of (peak-rest) V_E_/VCO_2_ association with HVS. Results were expressed as the odds ratios (OR) with 95% confidence intervals (CI). Decision statistics (2x2 tables) were calculated for several cut-off values of the difference of (peak-rest) P_ET_CO_2_ and the difference of (peak-rest) V_E_/VCO_2_. Data are summarized as mean ± SD or median (inter-quartile range) with p values <0.05 considered statistically significant. Statistical analysis was performed using Statistica 12.0 (StatSoft, Prague, Czech Republic).

## Results

Fifty-eight patients were included in this retrospective study. Twenty-nine patients were diagnosed with HVS and comprised the study group and 29 patients served as healthy controls. Group comparison of subject characteristics, pulmonary function test parameters and arterial blood gases are shown in Table 1. Subjects with HVS were mostly women with significantly lower BMI. There were no differences in pulmonary function test parameters or arterial blood gas analysis at rest. At peak exercise patients with HVS exhibited higher PaO_2_ and by definition higher pH and lower PaCO_2_.

**Table 1:** Group comparison.

	HVS (n = 29)	control (n = 29)	p
male No. (%)	4 (14)	14 (48)	<0.01
age (years)	56 (43–61)	61 (41–65)	0.77
height (cm)	168 (165–172)	170 (164–179)	0.23
BMI (kg/m^2^)	27.2 ± 5.8	30.5 ± 5.2	0.02
Pulmonary function test			
FEV_1_ (%)	95 (90–104)	100 (91–108)	0.30
FVC (%)	95 ± 11	97 ± 11	0.47
FEV_1_/FVC (%)	86 (83–93)	84 (81–89)	0.09
Arterial blood gas analysis at rest			
PaO_2_ (mmHg)	84 ± 9	83 ± 11	0.82
PaCO_2_ (mmHg)	36 (33–37)	36 (34–38)	0.44
BE	0.4 (-0.8–1.4)	0.1 (-0.5–0.9)	0.50
pH	7.45 (7.43–7.47)	7.44 (7.43–7.45)	0.07
Arterial blood gas analysis at peak exercise			
PaO_2_ (mmHg)	96 (93–109)	88 (83–93)	<0.01
PaCO_2_ (mmHg)	29 ± 4	35 ± 3	<0.01
BE	-2.0 ± 1.8	-2.9 ± 1.7	0.05
pH	7.47 (7.46–7.50)	7.40 (7.37–7.42)	<0.01

Data shown as mean ± SD or median (IQR). BE = base excess; cm = centimeter; FEV_1_ = forced expiratory volume-one second; FVC = forced vital capacity; HVS = hyperventilation syndrome; kg = kilogram; m^2^ = square meter; min = minute; ml = milliliter; mmHg = millimeter of mercury; PaCO_2_ = partial pressure of arterial carbon dioxide; PaO_2_ = partial pressure of arterial oxygen; VO_2_ = oxygen consumption

Rest and peak exercise ventilatory parameter comparison is shown in Table 2. At rest, the only difference between the study groups was in P_ET_CO_2_ which was significantly lower in patients with HVS. At peak exercise, patients with HVS also had significantly lower VO_2_, VCO_2_, V_T_ (including after correction for BSA) and P_ET_CO_2_ and higher f_b_, V_D_/V_T_, RER and V_E_/VCO_2_ ratio and slope.

**Table 2:** Cardiopulmonary exercise testing.

	HVS (n = 29)	control (n = 29)	p
rest			
VO_2_ (l/min)	0.32 (0.25–0.40)	0.38 (0.29–0.50)	0.29
VO_2_ (ml/kg/min)	4.2 (3.4–5.8)	4.3 (2.9–6.4)	0.91
VCO_2_ (l/min)	0.26 (0.19–0.31)	0.27 (0.21–0.36)	0.61
V_E_ (l/min)	10 (8–12)	11 (7–13)	0.96
V_T_ (l)	0.49 (0.41–0.65)	0.55 (0.46–0.76)	0.53
V_T_/BSA (l/m^2^)	0.27 (0.21–0.37)	0.29 (0.22–0.35)	0.91
f_b_ (bpm)	19 ± 5	18 ± 5	0.42
V_D_/V_T_	0.21 ± 0.11	0.22 ± 0.10	0.77
P_ET_CO_2_ (mmHg)	27 (25–30)	30 (28–32)	0.05
V_E_/VCO_2_ ratio	38 (33–44)	37 (32–40)	0.31
RER	0.76 (0.67–0.90)	0.69 (0.65–0.75)	0.08
HR (beat/min)	97 ± 13	82 ± 13	<0.01
peak exercise			
Workload (W)	135 (111–142)	163 (137–186)	<0.01
VO_2_ (l/min)	1.37 (1.30–1.72)	2.00 (1.59–2.47)	<0.01
VO_2_ (ml/kg/min)	18.7 (15.8–21.6)	24.2 (19.4–29.2)	0.01
VCO_2_ (l/min)	1.38 (1.24–1.57)	1.81 (1.56–2.15)	<0.01
V_E_ (l/min)	55 (46–62)	53 (48–63)	0.69
V_T_ (l)	1.25 (1.16–1.64)	1.87 (1.55–2.29)	<0.01
V_T_/BSA (l/m^2^)	0.72 (0.61–0.87)	1.01 (0.77–1.07)	<0.01
f_b_ (bpm)	39 (34–46)	30 (27–33)	<0.01
V_D_/V_T_	0.18 ± 0.05	0.13 ± 0.06	<0.01
P_ET_CO_2_ (mmHg)	27 ± 4	35 ± 4	<0.01
V_E_/VCO_2_ ratio	38 (35–43)	31 (27–34)	<0.01
RER	0.96 ± 0.1	0.89 ± 0.12	0.03
HR (beat/min)	142 ± 17	147 ± 20	0.30
V_E_/VCO_2_ slope	37 (33–43)	27 (24–30)	<0.01

Data shown as mean ± SD or median (IQR). bpm = breaths per minute; BSA = body surface area; f_b_ = breathing frequency; HR = hear rate; HVS = hyperventilation syndrome; kg = kilogram; l = liter; m^2^ = square meter; min = minute; ml = milliliter; mmHg = millimeters of mercury; P_ET_CO_2_ = partial pressure of end-tidal carbon dioxide; RER = respiratory exchange ratio; VCO_2_ = carbon dioxide output; V_D_ = dead space volume; V_E_ = minute ventilation; V_E_/VCO_2_ = ventilatory efficiency; VO_2_ = oxygen consumption; V_T_ = tidal volume; W = watts

**Fig 1:**
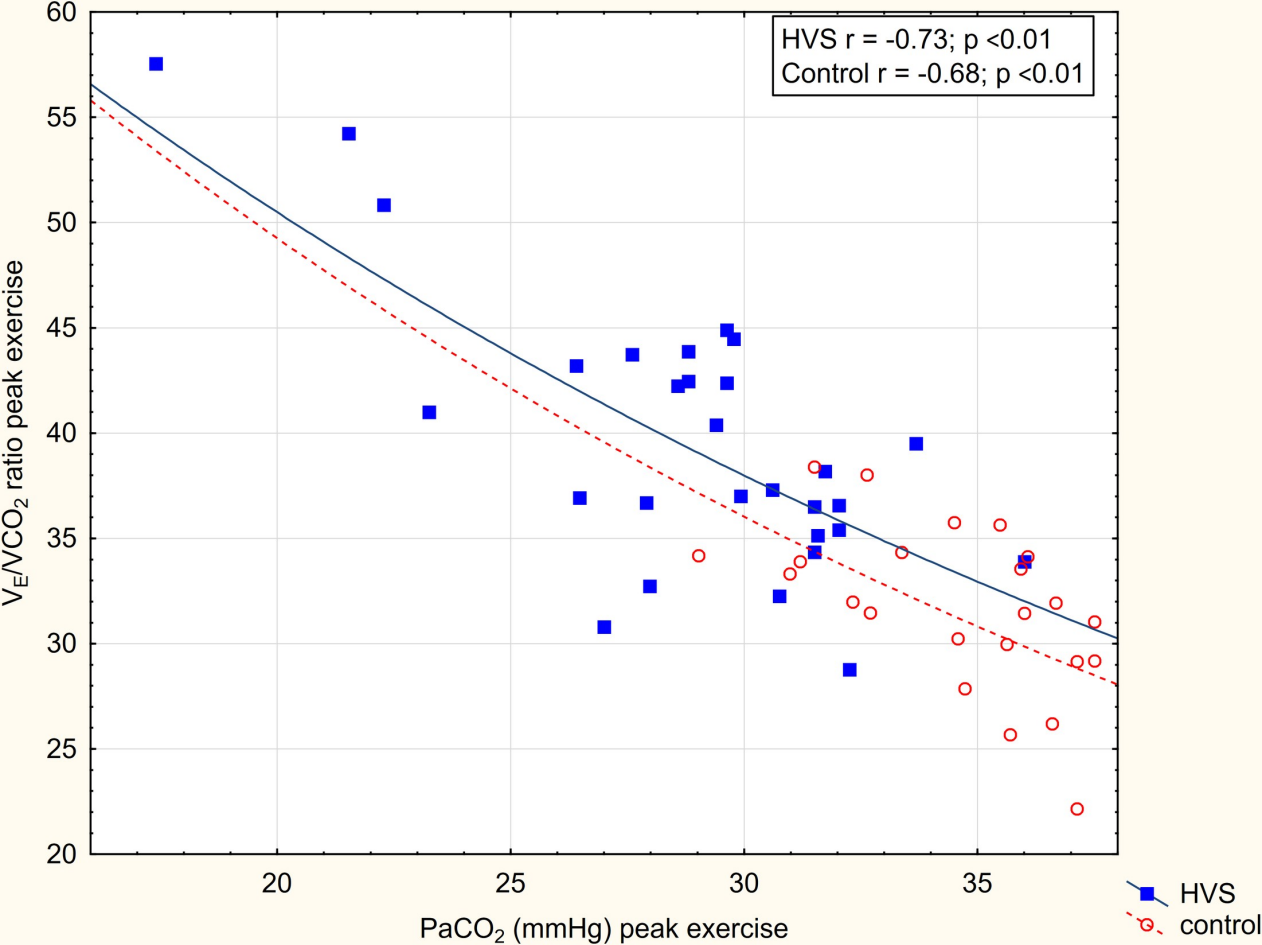
Relation of V_E_/VCO_2_ and PaCO_2_ in patients with HVS and controls. Slopes of ventilatory efficiency (V_E_/VCO_2_) to partial pressure of arterial oxygen (PaCO_2_) at peak exercise are compared in patients with HVS and controls. The shift of the slope of this relationship in patients with HVS is consistent with the observed higher V_D_/V_T_ ratio (i.e. higher ventilation-perfusion mismatch).

**Fig 2:**
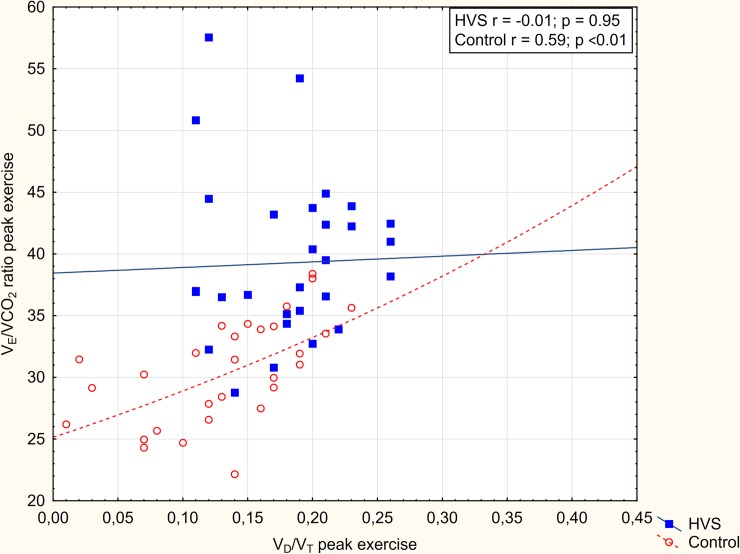
Relation of V_E_/VCO_2_ and V_D_/V_T_ in patients with HVS and controls. Slopes of V_E_/VCO_2_ and ratio of tidal volume to dead space (V_D_/V_T_) at peak exercise are compared in patients with HVS and controls. The shift of the slope of this relationship in patients with HVS is consistent with the observed lower PaCO_2_ (i.e. increased ventilatory drive).

The relation of V_E_/VCO_2_ ratio and PaCO_2_ at peak exercise in patients with HVS and in controls is shown in Fig 1. In both groups, there was a significant correlation of PaCO_2_ and the V_E_/VCO_2_ ratio. However, a shift of the slope of this relation was observed in patients with HVS. The shift is consistent with a significantly higher V_D_/V_T_ ratio (ventilation-perfusion mismatch) in patients with HVS. Similarly, Fig 2 shows the relation of V_E_/VCO_2_ ratio and V_D_/V_T_ at peak exercise in patients with HVS and in controls. The V_E_/VCO_2_ ratio correlated significantly with V_D_/V_T_ only for control subjects. In HVS subjects, the correlation was not observed and the slope shifted upwards. The shift of slope corresponds with the observed significantly lower PaCO_2_ consistent with increased ventilatory drive in patients with HVS.

**Fig 3:**
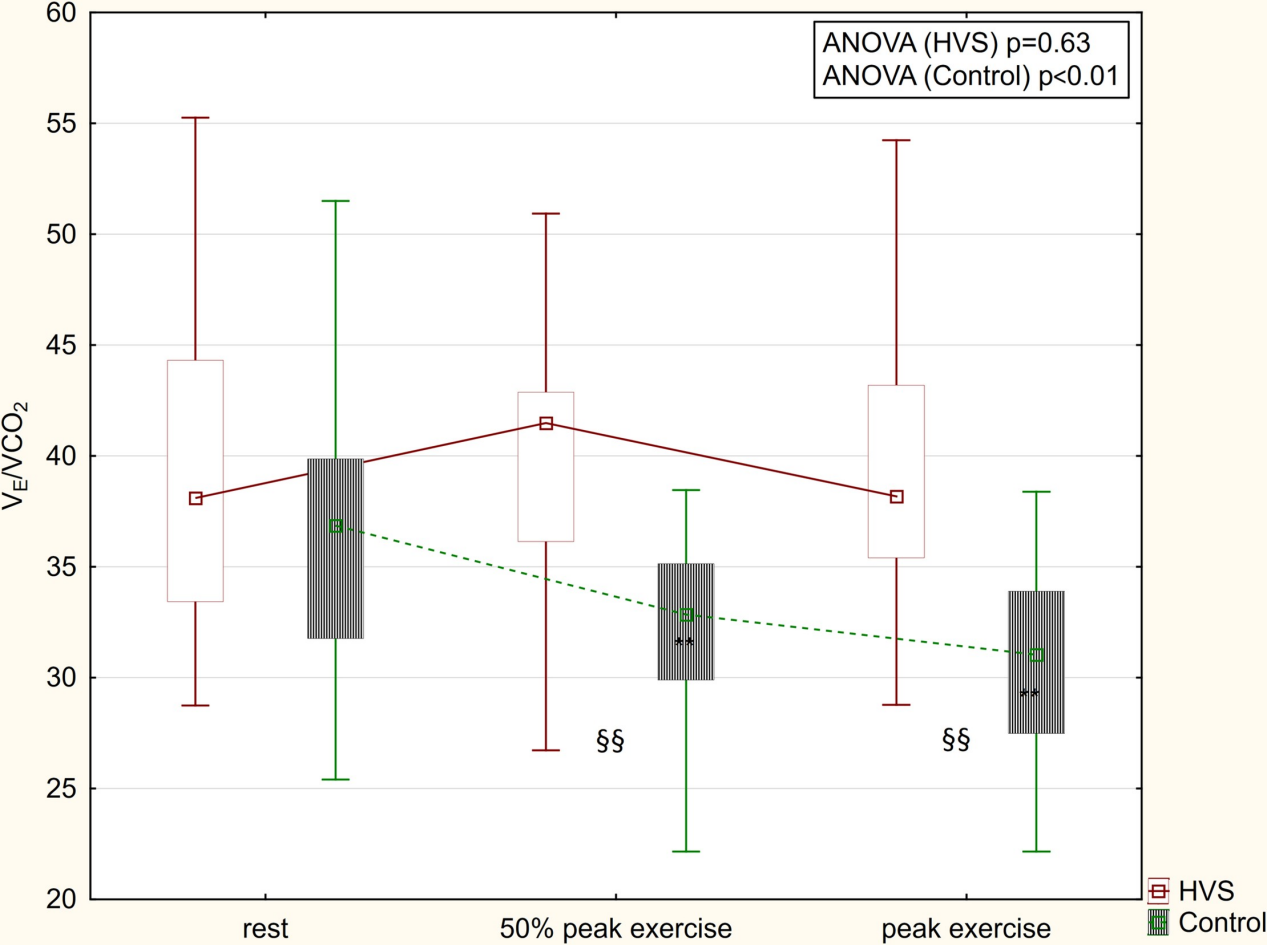
V_E_/VCO_2_ changes during exercise. In contrast to patients with HVS, V_E_/VCO_2_ decreased during exercise in controls. ** = p<0.01 compared to rest; §§ = p<0.01 HVS vs. control.

**Fig 4:**
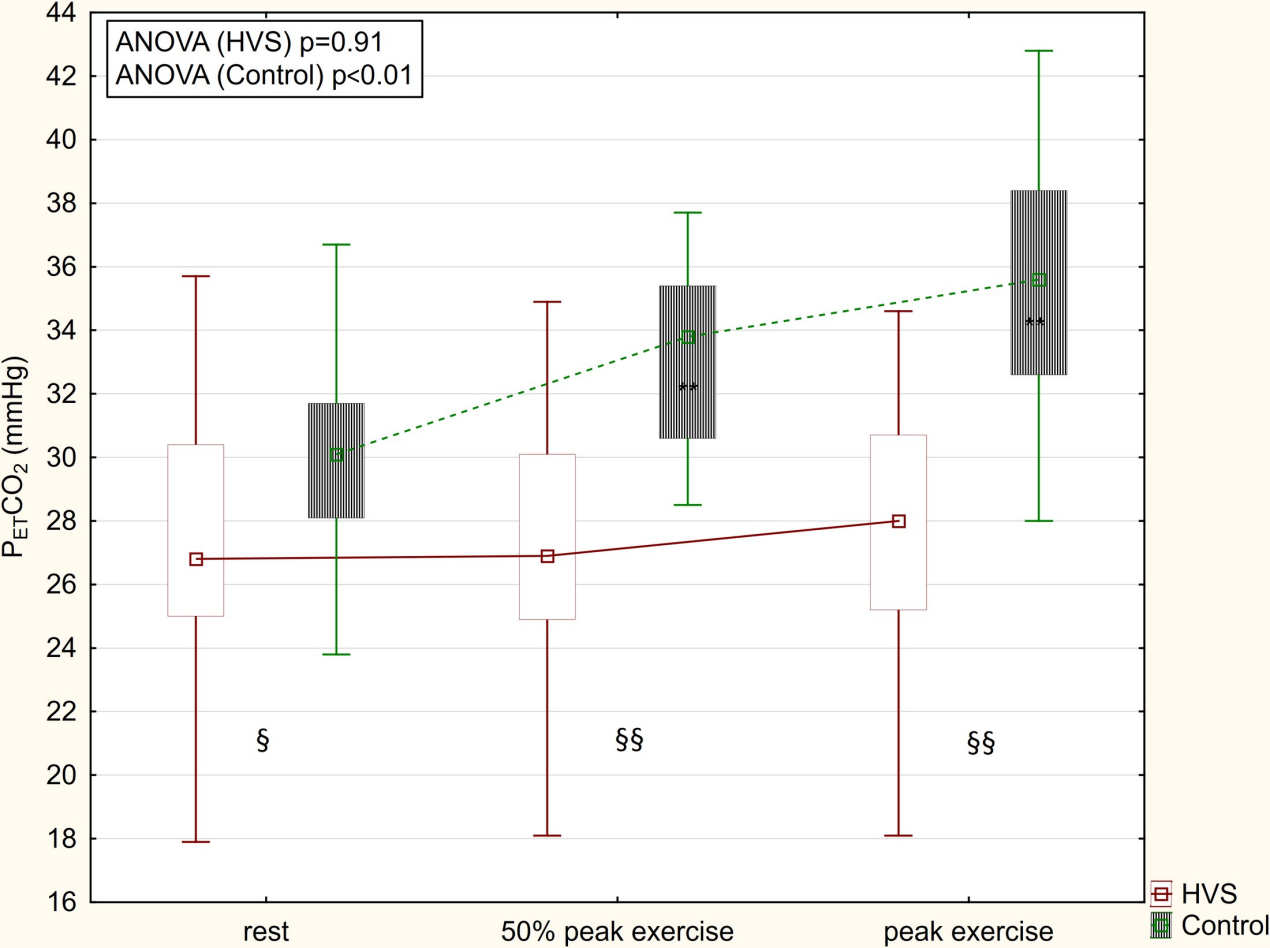
P_ET_CO_2_ changes during exercise. In contrast to patients with HVS, P_ET_CO_2_ increased during exercise in controls. ** = p<0.01 compared to rest; § = p<0.05 HVS vs. control; §§ = p<0.01 HVS vs. control.

Differences from peak exercise to rest of ventilatory parameters are summarized in Table 3. In patients with HVS, the increase of VO_2_, VCO_2_, V_T_ and heart rate (HR) during exercise was significantly lower than in controls. Breathing frequency increased significantly more in patients with HVS. In contrast to controls, V_E_/VCO_2_ and P_ET_CO_2_ did not change significantly during exercise in patients with HVS (Figs 3 and 4).

**Table 3:** Change of ventilatory parameters (peak-rest).

	HVS (n = 29)	control (n = 29)	p
VO_2_ (l/min)	1.10 (0.98–1.38)	1.65 (1.24–2.02)	<0.01
VO_2_ (ml/kg/min)	15.2 ± 6.0	20.4 ± 6.7	<0.01
VCO_2_ (l/min)	1.1 (1.0–1.3)	1.5 (1.3–1.8)	<0.01
V_E_ (l/min)	44 ± 14	43 (39–51)	0.70
f_b_ (bpm)	22 ± 8	12.2 ± 7.1	<0.01
V_T_ (l)	0.88 ± 0.36	1.36 ± 0.54	<0.01
V_D_/V_T_	-0.03 ± 0.09	-0.09 ± 0.11	0.04
P_ET_CO_2_ (mmHg)	0.50 ± 5.26	6.2 ± 4.6	<0.01
V_E_/VCO_2_ ratio	0.17 (-4.24–6.02)	-6.6 (-11.4-(-2.8))	<0.01
RER	0.18 (0.04–0.31)	0.2 (0.14–0.28)	0.69
HR (beat/min)	45 ± 14	65 ± 15	<0.01

Data shown as mean ± SD or median (IQR). bpm = breaths per minute; f_b_ = breathing frequency; FiO_2_ = fraction of inspired oxygen; HR = hear rate; HVS = hyperventilation syndrome; kg = kilogram; l = liter; min = minute; ml = milliliter; mmHg = millimeters of mercury; PaCO_2_ = partial pressure of arterial carbon dioxide; VCO_2_ = carbon dioxide output; V_D_ = dead space volume; V_E_ = minute ventilation; V_E_/VCO_2_ = ventilatory efficiency; VO_2_ = oxygen consumption; V_T_ = tidal volume

Logistic regression adjusted for gender and BMI showed the change of VO_2_ (OR 1.21; 95%CI 1.05–1.38; p = 0.01; ROC AUC = 0.84), VCO_2_ (OR = 24; 95%CI 1.8–318; p = 0.02; ROC AUC = 0.81), f_b_ (OR = 0.83; 95%CI 0.74–0.93; p<0.01; ROC AUC = 0.87), V_T_ (OR = 6.7; 95%CI 1.3–36; p = 0.03; ROC AUC = 0.79), HR (OR = 1.14; 95%CI 1.05–1.23; p<0.01; ROC AUC = 0.90), P_ET_CO_2_ (OR 1.26; 95%CI 1.08–1.48; p<0.01; ROC AUC = 0.83) and change of V_E_/VCO_2_ (OR 0.88; 95%CI 0.80–0.97; p = 0.01; ROC AUC = 0.81) to be independently associated with the presence of HVS. Decision statistics for several cut-off values of the change of V_E_/VCO_2_ and change of P_ET_CO_2_ and the diagnosis of HVS are shown in Table 4. The absence of V_E_/VCO_2_ or P_ET_CO_2_ changes during exercise was highly specific for HVS (83% and 93%, respectively). Specificity further increased with V_E_/VCO_2_ increase (up to 97%) and P_ET_CO_2_ decrease (up to 100%) during exercise.

**Table 4:** Decision statistics for the change of V_E_/VCO_2_ and P_ET_CO_2_ cut-off values and HVS.

V_E_/VCO_2_ ratio						
Δ (peak-rest)	sensitivity	specificity	+LR	-LR	PPV	NPV
+5	31 (15–51)	97 (82–100)	9 (1.2–67)	0.7 (0.6–0.9)	90 (55–99)	58 (52–64)
0	52 (33–71)	83 (64–94)	3 (1.3–7.2)	0.6 (0.4–0.9)	75 (56–88)	63 (53–72)
-5	83 (64–94)	59 (39–76)	2 (1.3–3.2)	0.3 (0.1–0.7)	67 (56–76)	77 (59–89)
-10	97 (82–100)	34 (18–54)	1.5 (1.1–1.9)	0.1 (0.01–0.73)	60 (53–66)	91 (58–99)
P_ET_CO_2_ (mmHg)						
Δ (peak-rest)	sensitivity	specificity	+LR	-LR	PPV	NPV
-5	21 (8–40)	100 (88–100)	-	0.8 (0.7–1)	100	56 (51–60)
0	55 (36–74)	93 (77–99)	8 (2–31)	0.5 (0.3–0.7)	89 (67–97)	68 (58–76)
+5	83 (64–94)	62 (42–79)	2.2 (1.3–3.6)	0.3 (0.1–0.7)	69 (57–78)	78 (61–89)
+10	93 (77–99)	14 (4–32)	1.1 (0.9–1.3)	0.5 (0.1–2.5)	52 (48–56)	67 (28–91)

Δ = delta; +LR = positive likelihood ratio; -LR = negative likelihood ratio; NPV = negative predictive value; P_ET_CO_2_ = partial pressure of end-tidal carbon dioxide; PPV = positive predictive value; V_E_/VCO_2_ = ventilatory efficiency

## Discussion

The major finding of this study was that in patients with HVS both the V_E_/VCO_2_ and P_ET_CO_2_ remained relatively unchanged from rest to peak exercise in patients with HVS consistent with abnormal ventilatory control throughout exercise.”

In our study, subjects with HVS were mostly women with lower BMI. This is in agreement with previous studies showing HVS to be more prevalent in women [6,19]. There were no significant differences in rest arterial blood gases between HVS subjects and controls. However, there was a nonsignificant trend towards higher pH in subjects with HVS. As there was no difference in PaCO_2_ at rest, we speculate higher pH may correspond with metabolic compensation of chronic episodes of hyperventilation in patients with HVS. Indeed, base excess tended to be higher in patients with HVS. At peak exercise, PaO_2_ was significantly higher and pH was significantly higher which exceeded 7.45 as per HVS definition and PaCO_2_ was significantly lower in patients with HVS compared to controls. Peak exercise arterial blood gases were very similar to values showed in a larger previous study [6].

At rest, P_ET_CO_2_ was significantly lower in subjects with HVS. However, logistic regression adjusted for confounders (gender and BMI) failed to show rest P_ET_CO_2_ to be significantly associated with the presence of HVS (OR 1.12; 95% CI 0.99–1.28; p = 0.08). In the Hammo et al. study [13], no difference was found in rest P_ET_CO_2_ in patients with HVS and controls. However, only 10 patients with HVS were included in this study [13], suggesting the study may have been underpowered. In contrast to our study, Kinnula et al. [12] showed significantly higher V_E_/VCO_2_ ratio at rest in patients with HVS. The V_E_/VCO_2_ ratio at rest was extremely high in the Kinnula et al. study (59±9.8) [12], suggesting a highly selected cohort. In contrast, a larger number of consecutive subjects with HVS included in our study may explain this apparent discrepancy.

At peak exercise, subjects with HVS exhibited lower VO_2_ which is in agreement with a previous study [20]. There was no difference in V_E_ between subjects with HVS and controls. However, the VCO_2_ was significantly lower in patients with HVS, suggesting an inadequately increased V_E_ for metabolic demand. Moreover, the breathing pattern was significantly different; f_b_ was significantly higher and V_T_ was significantly lower (even after correction for BSA) in subjects with HVS. Moreover, P_ET_CO_2_ was lower while V_D_/V_T_ and peak V_E_/VCO_2_ ratio were significantly higher in patients with HVS compared to controls. Our observation of increased peak V_E_/VCO_2_ ratio in patients with HVS is consistent with previous reports [12,20]. By the alveolar gas equation, V_E_/VCO_2_ is increased by either an increase of V_D_/V_T_ or by a decrease of PaCO_2_ [16]. In our subjects, we showed both an increase of V_D_/V_T_ (ventilation-perfusion mismatch) and a decrease of PaCO_2_ (increased ventilatory drive) contribute to the elevated V_E_/VCO_2_ ratio at peak exercise (Figs 1 and 2). We thereby confirm and extend the previous observations [12,20].

An increased V_E_/VCO_2_ ratio has been proposed as a diagnostic screen for HVS [12]. However, increased V_E_/VCO_2_ and exertional dyspnea are common in several conditions which need to be distinguished from HVS including heart failure [21], COPD [14], asthma [20], restrictive lung disease [22] and pulmonary artery hypertension (PAH) [23]. Therefore, we aimed to find a more specific marker of HVS. However, most of the other gas exchange and ventilatory parameters found to be significantly different in our HVS patients may also be associated with other conditions characterized by exertional dyspnea. Significant increase of f_b_ with diminished increase of V_T_ (i.e. rapid shallow breathing pattern) is frequent in heart failure and in both COPD and restrictive lung disease patients [24,25]. Diminished increase of VO_2_ during exercise may be seen in heart failure patients, especially in those with central sleep apnea (i.e., in those HF patients with the highest ventilatory drive) [24]. Only the (absence of changes) of V_E_/VCO_2_ and P_ET_CO_2_ during exercise were specific.

Physiologically, V_E_/VCO_2_ decreases and P_ET_CO_2_ increases from rest to peak exercise [26]. This physiological pattern may also be observed in patients with heart failure [24,27], COPD [28] and restrictive lung disease [28]. In contrast, in our subjects with HVS, the V_E_/VCO_2_ ratio and P_ET_CO_2_ did not change significantly from rest to peak exercise (Figs 3 and 4). Moreover, an inverse trend for increased V_E_/VCO_2_ and decreased P_ET_CO_2_ was highly specific for the presence of HVS (97% and 100%, respectively). Therefore, we speculate an increased V_E_/VCO_2_ combined with an inverse trend of V_E_/VCO_2_ and P_ET_CO_2_ during exercise may be helpful in the identification of subjects with HVS and in distinguishing these patients from other patients with exertional dyspnea caused by chronic heart failure, COPD and restrictive lung diseases. In asthma, the response to exercise may vary between patients and over time [29]. Therefore, making comparisons with asthmatic patients may be problematic.

PAH is also associated with exercise dyspnea and increased V_E_/VCO_2_ [23]. In patients with moderate-severe PAH, V_E_/VCO_2_ increases [30] and P_ET_CO_2_ decreases because of poor pulmonary perfusion during exercise [22]. In contrast to obstructive and restrictive lung diseases (heart failure is also characterized by both pulmonary restriction and obstruction [31]), in patients with PAH the ventilatory response to exercise seems to be more closely related to ventilatory-perfusion mismatch and increased ventilatory drive than to pulmonary mechanics [32]. Indeed, we have observed a similar inverse trend of V_E_/VCO_2_ and P_ET_CO_2_ in some of our HVS patients and showed its association with both ventilatory-perfusion mismatch and increased ventilatory drive, suggesting the ventilatory response to exercise in patients with PAH and HVS may be similar. However, it also suggests the inverse trend of ventilatory response to exercise may not allow discrimination of patients with HVS from those with PAH.

Our study has several limitations. First, it was a retrospective study. Second, only subjects with HVS and healthy controls were included. This study design allowed us to describe the HVS phenotype though it also prevents the generalization of these findings to the broader population. Third, there was a significant difference in RER between subject groups at peak exercise (RER was lower in controls). Lower RER may suggest lower exercise intensity in controls. However, the workload at peak exercise was significantly higher in controls, which further supports the concept of impaired ventilatory control resulting as inappropriately high ventilation in patients with HVS during exercise. Moreover, there was a trend towards lower RER at rest in controls also (p = 0.08) and there was no difference in the change of RER during exercise between both groups. We believe the lower RER might have been caused by inclusion of trained individuals (former athletes) as controls, as trained subjects have been shown to exhibit lower RER compared to untrained subjects at the same workload [33]. Fourth, P_ET_CO_2_ was lower than normal in both groups at rest. Resting values were obtained while sitting on the cycle ergometer with a facemask on. This might have caused a stress-related increase in minute ventilation and decrease of P_ET_CO_2_ in both groups. Indeed, psychological stress might have caused further increase in ventilation and may explain the even lower P_ET_CO_2_ in our subjects with HVS at rest (HR was significantly higher in the HVS group at rest) [4]. Fifth, HVS was defined as respiratory alkalosis–i.e., low PaCO_2_ by arterial blood gas analysis at peak exercise (similar to previous studies [6,7]). The use of low PaCO_2_ as a selection criterion for HVS patients may make the comparison of other peak exercise parameters related to PaCO_2_ (like P_ET_CO_2_ and V_E_/VCO_2_ ratio) difficult. However, V_E_/VCO_2_ has been shown to be influenced not only by PaCO_2_ (increased ventilatory drive), but also nearly equally by V_D_/V_T_ (ventilation-perfusion mismatch) [16]. Contributors to low P_ET_CO_2_ may also include both increased ventilatory drive and ventilation-perfusion mismatch. Indeed, peak exercise V_D_/V_T_ was significantly higher in our patients with HVS as was the alveolar-arterial difference of CO_2_ ((0 (-1.7–1.3) vs. -1.8 (-3.0–0.6); p = 0.03)). Therefore, as these two parameters (peak P_ET_CO_2_ and peak V_E_/VCO_2_ ratio) are not solely influenced by peak PaCO_2_, we believe the comparison is justified. Moreover, we suggest submaximal parameters (which were not used to define HVS) like rest to peak exercise changes of V_E_/VCO_2_ and P_ET_CO_2_ may be used to detect and discriminate HVS from other conditions with exertional dyspnea like chronic heart failure, COPD or restrictive lung diseases.

## Conclusion

In subjects with HVS, both V_E_/VCO_2_ and P_ET_CO_2_ remained unchanged from rest to peak exercise in patients with HVS suggesting abnormal ventilatory control. These findings may promote recognition of the HVS phenotype by evaluation of patients by CPET.

## Supporting information

S1 Table Dataset. Minimal dataset.
(XLSX)
